# The efficacy and safety of left bundle branch pacing combined with sacubitril/valsartan or enalapril in the treatment of elderly coronary heart disease with chronic heart failure

**DOI:** 10.12669/pjms.41.9.12557

**Published:** 2025-09

**Authors:** Chen Zhao, Huan Zhang, Juan Zhang, Kangli Chen

**Affiliations:** 1 Chen Zhao Department of Cardiovascular Medicine Ward 2, The Affiliated Hospital of Northwest University, Xi’An Third Hospital, Xi’An, Shaanxi Province Province 710021, P.R. China; 2 Huan Zhang Department of Cardiovascular Medicine Ward 2, The Affiliated Hospital of Northwest University, Xi’An Third Hospital, Xi’An, Shaanxi Province Province 710021, P.R. China; 3 Juan Zhang Department of Cardiovascular Medicine Ward 2, The Affiliated Hospital of Northwest University, Xi’An Third Hospital, Xi’An, Shaanxi Province Province 710021, P.R. China; 4 Kangli Chen Department of Cardiovascular Medicine Ward 2, The Affiliated Hospital of Northwest University, Xi’An Third Hospital, Xi’An, Shaanxi Province Province 710021, P.R. China

**Keywords:** Coronary heart disease, Chronic heart failure, Enalapril, Left bundle branch pacing, Sacubitril/valsartan

## Abstract

**Objective:**

Left bundle branch pacing (LBBP) is the best alternative to biventricular pacing (BiVP) and cardiac resynchronization therapy (CRT). This study compares the efficacy and safety of LBBP combined with sacubitril/valsartan and LBBP combined with enalapril in treating elderly coronary heart disease (CHD) patients with chronic heart failure (CHF).

**Methodology:**

This retrospective observational study included clinical records of elderly CHD patients with CHF who received LBBP treatment at The Affiliated Hospital of Northwest University from January 2022 to August 2024. Based on the postoperative treatment regimen, patients were divided into the LBBP & sacubitril/valsartan and the LBBP & enalapril groups. Levels of left ventricular end diastolic diameter (LVEDd), left ventricular ejection fraction (LVEF), QRS duration, N-terminal B-type natriuretic peptide precursor (NT proBNP), soluble growth stimulating gene 2 protein (sSt2), matrix metalloproteinase-9 (MMP-9) and the occurrence of adverse events were compared between two groups before and after treatment.

**Results:**

A total of 111 patients met the inclusion criteria. Of them, 53 patients comprised the LBBP & sacubitril/valsartan group and 58 patients comprised the LBBP & enalapril group. After treatment, levels of LVEDd, LVEF, NT proBNP, sSt2 and MMP-9 in the LBBP & sacubitril/valsartan group were significantly improved compared to those in the LBBP & enalapril group (all P<0.05). There was no statistically significant difference in the occurrence of adverse events between the two groups (both P>0.05).

**Conclusions:**

LBBP combined with sacubitril/valsartan is more effective in treating elderly CHD with CHF than LBBP combined with enalapril.

## INTRODUCTION

With the aging of the population, the incidence and mortality rates of coronary heart disease (CHD) and chronic heart failure (CHF) in elderly patients have been on the rise.^1^ Left bundle branch block (LBBB) is a common complication of CHD combined with CHF, often leading to asynchronous ventricular contractions (QRS wave widening, LVEF decrease).^2^,^3^ Therefore, correcting LBBB, synchronizing left and right ventricular contractions and improving heart pumping efficiency are key to relieving symptoms.^2^,^4^ Left bundle branch pacing (LBBP) is a novel pacing technique that evolved from His bundle pacing (HBP).^4^ LBBP provides a new option for patients requiring cardiac resynchronization therapy (CRT) due to the easier pacing across blocked sites and more ideal pacing parameters.^4^,^5^ A meta-analysis of 3004 patients by Yousaf et al.^5^ found that patients who received LBBP had better clinical outcomes than patients who received biventricular pacing (BVP), including better left ventricular ejection fraction, QRS duration, echocardiogram response, New York Heart Association functional classification, length of hospital stay and mortality rate.

Sacubitril/valsartan is a novel drug that combines a neprilysin inhibitor (sacubitril) and an angiotensin receptor inhibitor (valsartan) and significantly reduces the risk of cardiovascular death and hospitalization in CHF patients.^6^,^7^ A meta-analysis by Kang et al.^7^ reported that in patients with heart failure and CKD, the sacubitril/valsartan regimen significantly increased estimated glomerular filtration rate (eGFR), reduced blood pressure and NT proBNP, compared to irbesartan, valsartan and enalapril, providing cardiovascular and renal benefits. However, as a classic angiotensin-converting enzyme inhibitor, enalapril is also widely used in the treatment of CHF, exerting effects such as lowering blood pressure and reducing cardiac load.^8^,^9^ Tsutsui et al.^9^ found that HF patients receiving enalapril had lower adverse hypertension events than the sacubitril/valsartan group. Still, the risk of death and hospitalization was similar in both groups. Therefore, continuously optimizing the treatment of HF remains a challenge, especially in the elderly population. Compared with enalapril, which inhibits angiotensin-converting enzyme (ACE) and subsequently reduces angiotensin II production, sacubitril/valsartan combines an angiotensin receptor blocker (ARB) with a neprilysin inhibitor, thereby blocking the RAAS at the receptor level while enhancing natriuretic peptide activity. This dual mechanism offers additional benefits in terms of hemodynamic regulation, anti-fibrosis, and neurohormonal modulation.^6^,^7^

Based on our literature review, there is limited published evidence directly comparing sacubitril/valsartan and enalapril in the context of LBBP. This study therefore aims to provide preliminary insight into optimizing pharmacologic strategies following LBBP in elderly CHF patients.

## METHODOLOGY

This retrospective observational study included elderly CHD patients with CHF who received LBBP treatment in The Affiliated Hospital of Northwest University from January 2022 to August 2024. Based on the postoperative treatment regimen, patients were divided into the LBBP & sacubitril/valsartan and the LBBP & enalapril groups.

### Ethical approval:

The ethics committee of our hospital approved the study with the number 2025LL029; dated April 10, 2025.

### Inclusion criteria:

- Meets the diagnostic criteria for CHD with CHF.^1^,^2^
- Electrocardiogram confirms the presence of LBBB.^3^
- LVEDD≥60mm, LVEF%≤35%.
- New York Heart Association (NYHA) functional classification II-IV.
- Age ≥ 65 years old.
- Postoperative treatment with sacubitril/valsartan or enalapril.

### Exclusion criteria:

- Failed CRT.
- Severe coagulation dysfunction, infective endocarditis, etc.
- Other serious diseases such as severe liver and kidney dysfunction, malignant tumors, etc.
- The six months follow-up has not been completed.

### LBBP:

As described by Huang et al.,^10^ LBBP was performed using Select Secure (model 3830; Medtronic) pacing leads delivered through fixed curves or deflectable sheaths (C315HIS and C304His, Medtronic). A left subclavian vein access was established, the C315 sheath was delivered to the vicinity of the tricuspid annulus and the 3830 active fixation lead was introduced through the sheath. An electrophysiological recording system (PORTA-I, Jinjiang Electronics) was used to record intracardiac electrograms with a gain of ≥ 0.05 mV/mm and a paper speed of 100mm/s. After mapping the HB potential near the interventricular septum, the electrode tip was moved about 2 cm along the right ventricular septum towards the apex direction at the right anterior oblique position of 30 °. Unipolar pacing testing was performed at this location and the QRS wave site was found in lead V1 where the pacing electrocardiogram shows a “W” shape and fluctuates at the bottom of the QRS. The sheath was rotated counterclockwise to keep the wire tip perpendicular to the interventricular septum and provide sufficient support for the wire to screw into the interventricular septum. The wire was rotated clockwise at this location to enter the interventricular septum and changes in electrocardiogram and pacing impedance were monitored during the rotation process. When the pacing electrocardiogram gradually evolved from the pre-rotation LBBB morphology to the right bundle branch block (RBBB) morphology and the pacing QRS gradually narrowed with the peak time of left ventricular excitation (LVAT, the time limit for pacing stimulation nail to reach the peak of R wave in leads V5-V6), the unipolar pacing impedance was first gradually increased and then gradually decreased. The success of LBBP was determined according to the detailed criteria (see below). When the left correction threshold of LBBP tested was less than 1.0V/0.4ms, the sheath was cut, the lead was fixed and postoperative X-ray images were taken. The X-ray exposure time during the LBBP surgery stage was controlled at around 10 minutes.

The success criteria for LBBP correction of LBBB were as follows:

- Pacing electrocardiogram showing RBBB morphology.
- As the pacing voltage increases, LVAT suddenly shortens (usually ≥ 10ms) or remains constant and shortest at different pacing voltages.
- As the pacing output decreases, S-QRS onset exhibits equipotential and peak time remains unchanged, indicating a transition from NSLBBP to SLBBP.
- As the pacing output decreases, the peak time suddenly prolongs, indicating a transition from NSLBBP to left septal pacing.
- Patients in the LBBP & enalapril group were administered enalapril maleate tablets orally after surgery (specification: 5mg * 8 tablets * 2 tablets; Yangtze River Pharmaceutical Group Co., Ltd.), with an initial dose of 5mg once a day. The dose was adjusted to 10-40mg/day according to blood pressure conditions, 2-3 times a day.
- Patients in the LBBP & sacubitril/valsartan group received postoperative oral sacubitril/valsartan sodium tablets (specification 0.1 × 14 tablets/box; produced by Beijing Novartis Pharmaceutical Co., Ltd.), with an initial dose of 25mg, twice a day, adjusted according to blood pressure conditions. When blood pressure was greater than 90/60 mmHg, the dosage was increased to the maximum tolerated dose of 75mg twice daily. Both treatments were continued for six months. Patient demographics, medical history and current medication status were collected. LVEDd, LVEF%, QRS duration, NT proBNP, sSt2 and MMP-9 levels were recorded before and six months after treatment. The occurrence of adverse events such as palpitations, rash, dizziness, dry cough, etc., was collected.

### Statistical analysis:

Categorical variables were summarized as frequency (percentage), and continuous variables as mean ± standard deviation or median (interquartile range), depending on data distribution. Normality was assessed using the Shapiro-Wilk test. For between-group comparisons, chi-square or Fisher’s exact test was used for categorical variables, and independent t-test or Mann-Whitney U test for continuous variables. Analyses were performed using SPSS version 27.0 (IBM), with a two-sided P-value <0.05 considered statistically significant. As this was a retrospective observational study, no formal sample size calculation was conducted; patient enrollment was based on available eligible records during the study period.

## RESULTS

A total of 111 patients were ultimately included in this study. Among them, there were 58 males and 53 females, with a median age of 72 (68-75) years. A total of 49 patients had a history of hypertension and 29 had a history of diabetes; 35 patients were NYHA Class-II, 53 were Class-III and 23 were Class-IV. As shown in Table-I, there was no statistically significant difference in age, gender, smoking history, NYHA heart function classification and overall medication use between the two groups of patients (P>0.05).

**Table-I:** Comparison of baseline data between the two groups of patients.

Baseline data	LBBP & sacubitril/ Valsartan (n=53)	LBBP & enalapril (n=58)	χ^2^/Z/t	P
Male (yes), n(%)	29(54.7)	29(50.0)	0.247	0.619
Age (years), M(IQR)	70(68-74)	72(69-76)	-1.436	0.151
BMI (kg/m), mean±SD	23.5±3.2	22.5±2.6	1.803	0.074
Smoking (yes), n(%)	30(56.6)	28(48.3)	0.770	0.380
Hypertension (Yes), n(%)	21(39.6)	28(48.3)	0.841	0.359
Diabetes (yes), n(%)	15(28.3)	14(24.1)	0.249	0.618
NYHA cardiac function classification, n(%)			1.291	0.524
II	16(30.2)	19(32.8)		
III	28(52.8)	25(43.1)		
IV	9(17.0)	14(24.1)		
Combination therapy, n(%)				
ACEI/ARB	26(49.1)	31(53.4)	0.214	0.644
Beta-blocker	20(37.7)	26(44.8)	0.574	0.449
Spironolactone	21(39.6)	17(29.3)	1.308	0.253
Digoxin	24(45.3)	28(48.3)	0.100	0.752
Loop diuretics	28(52.8)	20(34.5)	3.798	0.051

Before treatment, there was no significant difference in LVEDd, LVEF and QRS duration levels between the two groups of patients (P>0.05). After treatment, the levels of LVEDd, LVEF and QRS duration in both groups improved significantly compared to before treatment. The LVEDd and LVEF of the LBBP & sacubitril/valsartan group were significantly better than those of the LBBP & enalapril group (P<0.05) (Table-II).

**Table-II:** Comparison of various indicators between two groups of patients before and after treatment.

Variables	LBBP & sacubitril/ valsartan (n=53)	LBBP & enalapril (n=58)	Z/t	P
***Before treatment***				
LVEDd(mm)	64(62-68)	64(62-69)	-0.214	0.831
LVEF (%)	30.1±3.2	29.2±3.8	1.462	0.147
QRS duration (ms)	178±12	180±15	-0.981	0.329
***After treatment***				
LVEDd(mm)	53.2±5.8	57.3±5.5^#^	-3.916	<0.001
LVEF (%)	46.2±3.9	43.8±6.0^#^	2.548	0.012
QRS duration (ms)	113±13	118±15^#^	-1.761	0.081

***Note:*** Compared to before treatment,

# P<0.05.

Pre-treatment levels of NT proBNP, sSt2 and MMP-9 were comparable in both groups (P>0.05). After treatment, the levels of NT proBNP, sSt2 and MMP-9 in both groups significantly improved and were significantly better in the LBBP & sacubitril/valsartan group than in the LBBLBBP & enalapril group (P<0.05) (Table-III). As shown in Table-IV, there was no statistically significant difference between the two groups in terms of the single adverse events and the total number of adverse reactions (P>0.05).

**Table-III:** Comparison of NT proBNP, sSt2 and MMP-9 between two groups.

Variables	LBBP & sacubitril/ valsartan (n=53)	LBBP & enalapril (n=58)	t/Z	P
Before treatment				
NT-proBNP (pg/mL)	1592±185	1634±203	-1.131	0.260
sSt2 (ng/mL)	60.1±9.0	61.7±9.4	-0.901	0.370
MMP-9 (ng/L)	404(355-475)	420.5(359-484)	-0.827	0.408
After treatment				
NT-proBNP (pg/mL)	363±51	520±71^#^	-13.222	<0.001
sSt2 (ng/mL)	40.5±7.6	49.6±8.6^#^	-5.908	<0.001
MMP-9 (ng/L)	132(105-157)	162.5(144-195)^#^	-4.730	<0.001

***Note:*** Compared to before treatment,

# P<0.05.

**Table-IV:** Adverse effects, n(%).

Adverse events	LBBP & sacubitril/ valsartan (n=53)	LBBP & enalapril (n=58)	χ^2^	P
palpitation	1(1.9)	2(3.4)	0.000	1.000*
rash	2(3.8)	5(8.6)	0.434	0.510*
dizzy	2(3.8)	4(6.9)	0.094	0.759*
dry cough	2(3.8)	3(5.2)	0.000	1.000*
Total number of Occurrences	7(13.2)	15(25.9)	2.791	0.095

* fisher’s Exact Test.

## DISCUSSION

While LBBP as a resynchronization technique has gained growing research attention, few studies to date have specifically addressed how different pharmacologic agents interact with this pacing modality. Our comparative analysis contributes to this underexplored intersection of pacing strategy and pharmacotherapy. This study showed that the combination of LBBP and sacubitril/valsartan significantly improved heart function and biomarkers in CHD with CHF compared to the combination of LBBP and enalapril. This result is consistent with the findings of Wang et al.^11^, which showed that the combination of LBBP and sacubitril/valsartan can more effectively improve cardiopulmonary function, reduce myocardial injury and improve quality of life in the treatment of CHF. However, this study employed a subgroup analysis of CHD with CHF in individuals aged 65 and above. Current guidelines recommend prioritizing LBBP in elderly patients due to the minimal trauma associated with the procedure, since LBBP is closer to physiological pacing, has stable pacing parameters and reduces the risk of arrhythmia.^12^,^13^

The study by Wu et al.^14^ also confirmed that LBBP is more effective in improving patient symptoms and heart function compared to BVP, similar to HBP, mainly because LBBP has a greater advantage in restoring physiological activation sequence and is closer to the normal electrical conduction mode of the heart.^12^,^14^ Although biventricular pacing can improve ventricular synchrony, it is not achieved through the natural conduction system.^15^,^16^ Studies have showed that sacubitril/valsartan, which can inhibit both the renin-angiotensin-aldosterone system and enkephalin enzyme, shows better performance in improving cardiac hemodynamics and inhibiting myocardial remodeling.^6^,^7^,^11^ Mechanistically, enalapril acts by inhibiting ACE and reducing angiotensin II, whereas sacubitril/valsartan blocks the angiotensin receptor and simultaneously inhibits neprilysin, resulting in elevated levels of natriuretic peptides. This dual inhibition not only reduces cardiac preload and afterload but also attenuates myocardial fibrosis, oxidative stress, and maladaptive neurohormonal activation.^17^ Furthermore, sacubitril/valsartan is associated with superior efficacy and safety in HF patients compared with enalapril.^17^,^18^ This study also showed that in the treatment of elderly (>65 years old) CHD patients with CHF, the LVEDd and LVEF% levels in the LBBP combined with sacubitril/valsartan group were significantly better than those in the LBBP & enalapril group, with no significant difference in adverse events between the two groups. Together with previous observational studies, these results suggest potential advantages of LBBP combined with sacubitril/valsartan over the LBBP & enalapril regimen.^11^,^17^,^18^

This study showed that the combination of LBBP and sacubitril/valsartan significantly improved biomarkers such as NT proBNP, sSt2 and MMP-9 compared to the combination of LBBP and enalapril. NT-proBNP is an important diagnostic and prognostic biomarker of CHF, while sST2 is a member of the interleukin-1 receptor family that reflects myocardial strain and inflammatory activity, and is associated with adverse cardiac remodeling and fibrosis. MMP-9, a matrix metalloproteinase, plays a key role in extracellular matrix degradation and ventricular remodeling, and its overexpression has been linked to progressive myocardial damage and dysfunction.^19^,^20^ The combination of LBBP and sacubitril/valsartan, thus, has a more significant effect in improving cardiac structure and function, inhibiting myocardial remodeling and inflammatory response. This is consistent with the research results of Chen et al.^19^ and Pieske et al.^21^ The possible mechanism of this effect may be related to the ability of sacubitril/valsartan to regulate the neuroendocrine system, reduce cardiac load and create a favorable internal environment for LBBP to restore cardiac electrical synchrony.^17^-^21^ LBBP improves the synchrony of heart contraction, enhances the pumping ability of the heart and also enhances the therapeutic effect of sacubitril/valsartan.^18^-^21^ In addition, this study did not include quality-of-life (QoL) measurements such as the Kansas City Cardiomyopathy Questionnaire (KCCQ) or the Minnesota Living with Heart Failure Questionnaire (MLHFQ), due to the retrospective nature of the dataset and the absence of standardized QoL assessments in the hospital’s electronic records during the study period. As patient-reported outcomes are essential for evaluating therapeutic value from the patient perspective, future prospective studies are encouraged to incorporate standardized QoL instruments.

In terms of safety, there was no statistically significant difference in the incidence and total number of adverse events such as palpitations, rashes, dizziness and dry cough between the two groups of patients. This is consistent with the results of Tsutsui et al.^9^ However, evidence suggests that LBBP combined with sacubitril/valsartan has advantages in terms of adverse events.^22^,^23^ The occurrence of palpitations may be related to changes in cardiac electrophysiological activity. When LBBP adjusts the sequence of cardiac excitation, it may affect the autonomic nervous system regulation of some patients, causing palpitations and necessitating adjustment of pacing parameters.^24^ In this study, both groups developed rashes, possibly related to decreased physical function in the elderly or concomitant medication. Dizziness is often caused by unstable blood pressure or insufficient cerebral blood supply.^25^ As patients with CHD and CHF have poor cardiovascular function, the impact of drugs on blood pressure and changes in cardiac pumping function may induce dizziness and will require adjusting medication dosage as necessary to improve dizziness symptoms. Dry cough is a common adverse reaction of enalapril, caused by its inhibition of bradykinin degradation, leading to the accumulation of bradykinin.^26^ It has been shown that the incidence of cough can reach 10.5% after conventional enalapril treatment.^27^ However, in this study, there was no significant difference in the incidence of dry cough between the two groups, which may be due to the small sample size or the masking of adverse drug reactions by the effect of LBBP on cardiac hemodynamics. Additionally, underreporting of mild symptoms in retrospective medical records or the concomitant use of medications that alleviate cough (e.g., antitussives or mucolytics) may have further contributed to the low observed incidence. This possibility should be considered when interpreting the findings. This indicates that both combination therapy regimens have good safety in clinical application. However, due to the retrospective nature of the study and incomplete laboratory data, we were unable to assess specific safety indicators such as hyperkalemia, hypotension episodes, and renal function changes. These are clinically relevant adverse events associated with sacubitril/valsartan and enalapril. We recommend that future prospective studies include detailed biochemical monitoring to provide a more comprehensive safety profile. Although LBBP combined with sacubitril/valsartan has significant advantages in efficacy, its cost is relatively high and enalapril remains the first choice for many patients.^28^

In summary, this study provides real-world comparative evidence on two commonly used pharmacologic regimens in elderly patients with CHD and CHF undergoing LBBP. Its strengths lie in the targeted evaluation of a relatively underexplored patient population, the use of clinically meaningful biomarkers and echocardiographic parameters, and the focus on a practical therapeutic question. The findings add to the emerging body of knowledge supporting the integration of optimized pacing techniques with individualized medical therapy in heart failure management. Despite its limitations, this study offers preliminary guidance for clinical decision-making and highlights the need for larger, prospective trials in this area.

### Limitations:

It includes a single-center retrospective study with a relatively small sample size and lack of randomization, which may lead to selection bias and intergroup heterogeneity. Second, the short follow-up period of six months limits the evaluation of long-term structural and functional cardiac outcomes. Moreover, critical clinical endpoints such as cardiovascular mortality and heart failure rehospitalization were not included due to data limitations. Third, we were unable to conduct subgroup analyses based on renal function because of incomplete eGFR data, despite the known influence of renal status on the efficacy of sacubitril/valsartan. Finally, specific adverse events such as hyperkalemia, hypotension, and renal impairment were not systematically monitored in the medical records. These limitations highlight the need for future large-scale, multicenter, prospective studies with extended follow-up and comprehensive endpoint evaluation.

## CONCLUSION

This study demonstrates that LBBP combined with sacubitril/valsartan was associated with more favorable improvements in cardiac function and biomarker profiles compared to LBBP with enalapril, without an increase in adverse events. Given its enhanced efficacy and comparable safety, this combination may be considered a preferred treatment option for elderly CHF patients who can tolerate the medication and afford its cost. Further multicenter randomized controlled trials with extended follow-up are warranted to validate these findings and evaluate long-term clinical benefits.

### Authors’ Contributions:

**CZ:** Study design and manuscript writing. **HZ, JZ and KC:** Data collection, data analysis and interpretation. Critical analysis. **CZ:** Manuscript revision and validation and is responsible for the integrity of the study. All authors have read and approved the final manuscript.
